# *Allobaculum mucilyticum*-Mediated Gut Barrier Dysfunction Exacerbates the Severity of Hypertriglyceridemic Acute Pancreatitis in Mice

**DOI:** 10.3390/antiox14111284

**Published:** 2025-10-27

**Authors:** Ping Yang, Meirong Wu, Wenjie Liang, Yudong Sun, Li-Long Pan, Jia Sun

**Affiliations:** 1 School of Food Science and Technology, Jiangnan University, Wuxi 214122, China; 7180112049@stu.jiangnan.edu.cn (P.Y.);; 2 State Key Laboratory of Food Science and Resources, Jiangnan University, Wuxi 214122, China; 3 MOE Medical Basic Research Innovation Center for Gut Microbiota and Chronic Diseases, Wuxi School of Medicine, Jiangnan University, Wuxi 214122, China

**Keywords:** hypertriglyceridemic acute pancreatitis, gut dysbiosis, oxidative stress, mucus barrier dysfunction, M1 macrophages

## Abstract

Hypertriglyceridemic acute pancreatitis (HTGAP) is characterized by frequent severe complications and poor clinical prognosis. Recent evidence suggests that gut dysbiosis is correlated with pancreatic injury in HTGAP, although the precise mechanisms remain to be elucidated. Here, we found that experimental HTGAP mice exhibited gut dysbiosis and intestinal barrier dysfunction, accompanied by an abnormal increase in *Allobaculum mucilyticum* (*A. mucilyticum*) and a decrease in *Akkermansia muciniphila* (*A. muciniphila*). Administration of *A. mucilyticum* aggravated oxidative stress-associated intestinal barrier dysfunction, promoted bacterial translocation to the pancreas, and ultimately exacerbated pancreatic injury. Conversely, supplementation with *A. muciniphila* alleviated the severity of HTGAP by restoring mucus layer thickness and reducing intestinal pro-inflammatory macrophage polarization. These findings highlight the critical role of gut dysbiosis in HTGAP progression, mediated through the pro-inflammatory mucolytic pathobiont *A. mucilyticum*, and suggest that modulating gut microbiota may represent a novel therapeutic strategy for HTGAP.

## 1. Introduction

Acute pancreatitis (AP) is a potentially fatal gastrointestinal disorder that often requires emergency hospitalization. Its incidence is over 34 affected individuals per 100,000 of the general population annually and continues to rise worldwide [1]. Recently, abnormal lipid metabolism-induced hypertriglyceridemia (HTG) has overtaken alcohol abuse to become the second leading cause of AP following gallstones in China [2]. HTG initiates AP and further intensifies its severity [3,4,5,6]. Compared to other etiologies, the clinical course and outcomes of HTG-induced AP are more severe, with a higher recurrence rate and elicit serious associated complications such as infected pancreatic necrosis (IPN), systemic inflammatory response (SIR), and persistent multiple organ failure (MOF) [7,8,9]. Thus, elucidating the relevant pathological mechanisms by which HTG worsens AP will provide fresh insights and potential therapeutic strategies for ameliorating hypertriglyceridemic acute pancreatitis (HTGAP).

The initial stages of AP typically involve local aseptic inflammation driven by the infiltration of innate immune cells, which are recruited by damage-associated molecular patterns released from injured pancreatic acinar cells [10,11,12]. The inflammatory response is subsequently amplified by the translocation of enteropathogenic organisms to the pancreas following disruption of intestinal homeostasis, thereby exacerbating AP progression [10,13]. Specifically, the excessive infiltration and activation of pancreatic macrophages, polarized toward the M1 phenotype in response to local pro-inflammatory stimuli such as translocated intestinal pathogens or lipopolysaccharide (LPS), mediate and intensify the local secondary pancreatic inflammatory cascade by secreting large amounts of pro-inflammatory cytokines and chemokines [1,11,12]. Ultimately, this process results in an intractable SIR or MOF. Therefore, increased intestinal permeability caused by gut microbiota dysbiosis, intestinal barrier malfunction, and gut immunological imbalance is a critical determinant in driving the IPN of severe AP and linking the gut-pancreas axis [10,11,12,13,14,15,16,17,18].

Altered gut microbiota composition in HTGAP patients and experimental animal models plays a causal role in the aggravation and poor clinical prognosis of HTGAP [19,20,21]. Recent research demonstrated that the absence of a specific beneficial microbe and its metabolite in HTGAP promotes the excessive formation of neutrophil extracellular traps in the pancreatic region, thereby accelerating pancreatic injury [21]. Notably, this literature also observed a correlation between enhanced pancreatic macrophage infiltration and gut microbiota remodeled by HTG, although the underlying mechanisms were not investigated further. Furthermore, evidence indicates that disrupted intestinal flora contributes to the progression of HTGAP by reducing the secretion of antimicrobial peptides (AMPs) from Paneth cells in the rat ileum [19]. Nonetheless, limited information is available regarding which particular bacterial species contribute to the pathophysiological changes in HTGAP by compromising the gut barrier integrity and driving immune abnormalities within the intestinal mucosa and pancreas.

This study aims to investigate how dysregulated gut microbiota exacerbates the pathogenesis of HTGAP. We employed a broad-spectrum antibiotic (ABX) depletion strategy to validate the pathogenic effects of specific strains abnormally enriched in HTGAP. Our data clarify the exact role of this bacterial taxon in promoting pancreatic damage via mucus degradation and pro-inflammatory immune stimulation. Leveraging the competitive antagonism between pathogenic and commensal bacteria within ecological and immune niches, we propose a probiotic-based intervention strategy as a promising avenue to mitigate the adverse effects of HTGAP.

## 2. Materials and Methods

### 2.1. Mice

All animal experimental procedures were conducted in compliance with the ARRIVE guidelines for preclinical studies [22] and approved by the Animal Ethics Committee of Jiangnan University (Approval No.: JN. No 20230915m0720330[415]). Male C57BL/6 mice aged 6–8 weeks used in this research were purchased from GemPharmatech (Nanjing, China) and housed in groups of 4–5 per individually ventilated cage under specific pathogen-free conditions. Animals were maintained on a 12 h light/12 h dark cycle at 22 ± 2 °C and 60 ± 5% relative humidity, with free access to standard laboratory diet and water ad libitum. Animals were randomly and blindly assigned to groups of equal size (*n* = 6). Vehicle and treatment groups were kept in separate cages to prevent cross-contamination among the different experimental groups.

### 2.2. Animal Model

HTGAP was induced by poloxamer 407 (P407, P2443, Sigma-Aldrich, St. Louis, MO, USA) in conjunction with caerulein (CER, S62702, Yuanye Biotechnology, Shanghai, China) as previously described [3,4,6,21]. Mice in the HTGAP group were intraperitoneally injected with the P407 solution (dose: 0.5 g/kg) every second day for 28 days, after first mixing 0.5 g of P407 with 10 mL of 0.01 M sterilized phosphate-buffered saline (PBS, pH = 7.4) and refrigerating at 4 °C overnight to dissolve completely. Mice assigned to the control and AP groups received an equivalent volume of PBS in the same manner. Subsequently, 10 intraperitoneal injections of CER (dose: 50 μg/kg) were administered to mice in the AP and HTGAP groups at one-hour intervals. An identical amount of PBS was given to the control group simultaneously.

All mice were sacrificed 12 h after the initial injection of CER. Serum triglyceride (TG) and total cholesterol (TC) concentrations were measured using Beckman Coulter AU chemistry analyzers to confirm the successful induction of HTG. Enzyme activity assay kits were utilized to detect serum amylase (C016-1-2, Nanjing Jiancheng, Nanjing, China) and lipase (A054-2-1, Nanjing Jiancheng, Nanjing, China), the clinical diagnostic indicators of AP, to verify the effective establishment and severity of AP.

### 2.3. Pancreatic Edema and Myeloperoxidase Activity

Pancreatic edema was quantified by the ratio of wet weight to dry weight. The initial weight of the freshly harvested pancreatic tissue was defined as the wet weight. The weight of the identical sample after desiccation at 60 °C for 72 h was used as the dry weight. Pancreatic myeloperoxidase (MPO) activity was measured using an MPO activity assay kit (BC5715, Solarbio, Beijing, China) according to the manufacturer’s instructions.

### 2.4. Histopathological Analysis

Fresh pancreatic and colonic tissues were fixed in 4% paraformaldehyde and embedded in paraffin. Slices of 4 μm thickness were annealed to slides by heating at 60 °C for 1 h and rehydrated using a graded ethanol series after being dewaxed twice (10 min, each) by immersion in xylene. Sections were stained with hematoxylin and eosin (H&E) and scanned under a Pannoramic MIDI Scanner (3DHISTECH, Budapest, Hungary).

Histological scores were assessed blindly by two trained pathologists. Pancreatic pathology was evaluated based on three categories: edema, inflammatory cell infiltration, and acinar cell necrosis [3]. The histopathological scores of colonic inflammation were assessed by the following two criteria: inflammatory cell infiltrates (Score 0, none; Score 1, low density limited to the mucosa; Score 2, moderate to high density in mucosa and/or low to moderate density in mucosa and submucosa; Score 3, high density in submucosa and/or extension to muscularis mucosae; Score 4, high density with frequent transmural extension) and mucosal epithelial architecture damage (Score 0, no abnormality; Score 1, mild goblet cell loss; Score 2, moderate goblet cell loss with cryptitis; Score 3, marked goblet cell loss with crypt abscesses; Score 4, Irregular crypts or crypt loss and surface epithelial destruction) [23]. An overall score was obtained by summing the scores assigned to each criterion.

### 2.5. Western Blot Analysis

Total protein was extracted using RIPA Lysis Buffer (P0013B, Beyotime Biotechnology, Shanghai, China) supplemented with 1% protease and phosphatase Inhibitor Cocktail (PR20016 and PR20015, Proteintech, Wuhan, China). Utilizing the BCA Protein Assay Kit (MA0082, Meilunbio, Dalian, China) to quantify the protein concentrations, approximately 40 μg of proteins were separated via 6–12% sodium dodecyl sulfate-polyacrylamide gel electrophoresis and transferred onto nitrocellulose membranes. Following an hour at room temperature (RT) blocking with 5% skim milk in Tris-buffered saline containing 0.1% Tween-20, the membranes with bands were incubated with specific primary antibodies (Table 1) overnight at 4 °C. Afterward, the blots were exposed to appropriate secondary antibodies (Table 2) for 1.5 h at RT and visualized with the ChemiDocTouch imaging system (Bio-Rad, Hercules, CA, USA) using a chemiluminescent reagent (Merck Millipore, Burlington, MA, USA). The protein levels were normalized to β-Actin or GAPDH and quantified using ImageJ (Fiji, version 1.53, National Institutes of Health, Bethesda, MD, USA) image processing software. The antibodies used in this study are listed in Table 1 and Table 2.

### 2.6. Immunofluorescence

Paraffin-embedded sections of the pancreas and colon were deparaffinized in xylene and rehydrated through a graded alcohol series. Heat-mediated antigen retrieval was performed using sodium citric buffer (P0083, Beyotime Biotechnology, Shanghai, China), followed by natural cooling. After PBS washes, sections were blocked with QuickBlock™ blocking buffer (P0260, Beyotime Biotechnology, Shanghai, China) for 1 h at RT and incubated with fluorochrome primary antibodies (Table 1) overnight at 4 °C. Slides were rinsed thrice with PBS and incubated in fluorochrome secondary antibody solution (Table 2) for 1.5 h at RT. Following another PBS wash, sections were counterstained with DAPI (C1006, Beyotime Biotechnology, Shanghai, China) for 8 min and mounted with Antifade Mounting Medium (P0126, Beyotime Biotechnology, Shanghai, China). Fluorescence images were acquired using a Carl Zeiss Axiolab 5 fluorescence microscope (Zeiss, Oberkochen, Germany) and processed for brightness and contrast correction, cropping, and addition of scale bars with Zen 3.4 software (Zeiss, Oberkochen, Germany). The integrated optical density values or the average number of fluorescently labeled positive cells were calculated using ImageJ software in six randomly selected regions. The antibodies used in this study are listed in Table 1 and Table 2.

### 2.7. Enzyme-Linked Immunosorbent Assay (ELISA)

To determine the levels of TNF-α (E-EL-M3063), IL-1β (E-EL-M0037), IL-6 (E-EL-M0044), and MCP-1 (E-EL-M3001) in pancreatic tissue, samples were homogenized in PBS and centrifuged at 10,000× *g* for 15 min at 4 °C. The supernatants were collected and analyzed using commercial ELISA kits (Elabscience Biotechnology, Wuhan, China) according to the manufacturer’s guidelines.

A substantial rise in serum LPS levels implies increased intestinal permeability and leaky gut, as the macromolecular endotoxin LPS cannot cross an intact intestinal barrier to enter the circulation. Mouse blood samples were collected and allowed to stand at RT for 1 h. Serum was separated by centrifugation at 3000× *g* for 20 min and analyzed using a mouse LPS ELISA kit (MM-0634M1, Meimian, Yangzhou, China) under the manufacturer’s instructions.

### 2.8. Fluorescence In Situ Hybridization (FISH)

After roasting at 62 °C for 2 h, the 4 μm Paraffin sections were dewaxed in xylenes, followed by gradient ethanol dehydration, and thoroughly dried before hybridization. Bacterial probe EUB338 (5′-Cy3-GCTGCCTCCCGTAGGAGT-3′) was diluted in sterile hybridization buffer (0.9 M NaCl + 0.02 M Tris-HCl, pH 7.5 + 20% Formamide + 0.05% SDS) to a final concentration of 1 μM and stained overnight in a humidified chamber at 46 °C. After rinsing with preheated hybridization wash solution (5 M NaCl + 0.02 M Tris-HCl, pH 7.5 + 5 mM EDTA + 0.01% SDS), slides were mounted with an Antifade Mounting Medium containing DAPI, and pictures were captured by a Carl Zeiss Axiolab 5 fluorescence microscope (Zeiss, Oberkochen, Germany) [18,24].

### 2.9. Measurement of Antioxidant Enzyme Activity and Lipid Peroxidation

10% mouse colonic tissue homogenate was used to measure superoxide dismutase (SOD) activity using an SOD assay kit (A001-3-2, Nanjing Jiancheng, Nanjing, China) based on the WST-1 method, and malondialdehyde (MDA) levels were determined using a microscale MDA assay kit (A003-2-2, Nanjing Jiancheng, Nanjing, China) based on the TBA method. SOD activity reflects the antioxidant capacity of the sample, while MDA levels indicate the degree of oxidative damage.

### 2.10. Alcian Blue-Periodic Acid-Schiff Staining

Alcian Blue-Periodic Acid-Schiff (AB-PAS) staining was employed to observe and differentiate the distribution and quantity of neutral and acidic mucins in colonic tissue mucopolysaccharides. Acidic mucus substances are first specifically dyed blue by 1% Alcian Blue, while neutral mucins remain uncolored at this stage. Subsequently, hydroxyl groups on adjacent carbon atoms in the glycogen and neutral mucins are oxidized into aldehyde groups by a 0.5% periodic acid solution, which is then combined with Schiff reagent to form magenta complexes. In light of this, mixed mucus substances are stained to various degrees of bluish-purple coloration.

### 2.11. RNA Isolation and Sequencing Analysis

After harvesting the pancreatic and colonic tissues from the mice, the colon was sliced longitudinally and thoroughly washed with sterile PBS until no visible fecal debris remained. Total RNA was extracted from six biological replicates per group using RNAiso Plus reagent (9109, Takara, Shiga, Japan) according to the manufacturer’s protocol. The concentration and quality of RNA were measured with a NanoPhotometer (N60 Touch, IMPLEN, Munich, Germany) and agarose gel electrophoresis. Library construction and sequencing were performed by HonsunBio Technology (Shanghai, China) on the Illumina NovaSeq 6000 platform, generating 150 bp paired-end (PE150) reads.

### 2.12. 16S rRNA Sequencing and Data Analysis

Microbial genomic DNA was extracted from mouse colonic contents using the FastDNA spin kit for feces (6570200, MP Biomedicals, Irvine, CA, USA). The concentration and purity of the extracted bacterial DNA were measured using a NanoPhotometer (N60 Touch, IMPLEN, Munich, Germany). The hypervariable V3-V4 region of bacterial 16S rRNA gene was amplified with barcoded fusion primers 341F (5′-CCTAYGGGRBGCASCAG-3′) and 806R (5′-GGACTACNNGGGTATCTAAT-3′). The PCR mixtures contained 25 μL of 2 × Phanta Flash Master Mix (P520, Vazyme, Nanjing, China), 0.2 μmol of each primer, and 10 ng target DNA. The cycling conditions consisted of an initial denaturation step at 95 °C for 5 min, followed by 30 cycles of 95 °C (30 s), 50 °C (30 s), and 72 °C (30 s), with a final extension at 72 °C for 7 min. These PCR products were then subjected to electrophoresis on a 1.5% agarose gel and purified using the DNA Gel/PCR Purification Miniprep Kit (BW-DC3511, BEIWO, Shanghai, China). Sequencing libraries were constructed and sequenced on an Illumina MiSeq PE300 platform.

The DADA2 algorithm implemented in QIIME2 was used to quality-filter, denoise, and remove chimeras from the raw DNA sequences, yielding initial amplicon sequence variants (ASVs). Species annotation was performed for all representative sequences of ASVs using the sklearn classifier through comparison to the Silva database. Alpha diversity, reflecting microbial community richness and evenness, was assessed using the Shannon index. Beta diversity was estimated by the ANOSIM method of the Bray–Curtis non-metric multidimensional scaling (NMDS) to explore the degree of discrepancy in bacterial community structures among groups. Differentially abundant bacterial taxa between groups were identified using linear discriminant analysis effect size (LEfSe) with a logarithmic linear discriminant analysis (LDA) score threshold of 4.

### 2.13. Bacterial Supplementation

*Allobaculum mucilyticum* (*A. mucilyticum*, DSM No. 112815) was obtained from Deutsche Sammlung von Mikroorganismen und Zellkulturen (DSMZ, Braunschweig, Germany) and anaerobically grown in DSMZ104 medium or on Columbia blood agar plates. *Akkermansia muciniphila* (*A. muciniphila*, ATCC BAA-835) was acquired from the American Type Culture Collection (ATCC, Manassas, VA, USA) and cultured anaerobically in brain heart infusion medium at 37 °C. The identity of each bacterial strain was confirmed at the species level by sequencing the V4 region of the 16S rRNA gene. Before use, bacteria were collected by centrifugation at 8000× *g* for 10 min and resuspended in sterile PBS.

The gut microbiota was depleted using an antibiotic treatment. In brief, mice received 200 μL of autoclaved water containing metronidazole (1 g/L), gentamycin (1 g/L), vancomycin (0.5 g/L), ampicillin (1 g/L), and Neomycin (1 g/L) via oral gavage daily for ten days [25]. *A. mucilyticum* (5 × 10^8^ CFU/200 μL per mouse) was administered by oral gavage every other day for 2 weeks, whereas *A. muciniphila* (5 × 10^8^ CFU/200 μL per mouse) was administered daily for 4 weeks [26]. An equivalent volume of sterile PBS was given as a vehicle control.

*A. mucilyticum* and *A. muciniphila* abundance were quantified using real-time qPCR with the Taq Pro Universal SYBR qPCR Master Mix (Q712, Vazyme, Nanjing, China) on a CFX Connect Real-Time System (Bio-Rad, Hercules, CA, USA). The analysis was conducted using species-specific primers for *A. mucilyticum* (F: 5′-AGCAGAAAGGAAATGGTCTG-3′, R: 5′-GTTTCCAAAGCCTGCACAGG-3′), *A. muciniphila* (F: 5′-CAGCACGTGAAGGTGGGGAC-3′, R: 5′-CCTTGCGGTTGGCTTCAGAT-3′), and the universal bacterial 16S rRNA gene (F: 5′-GCAGGCCTAACACATGCAAGTC-3′, R: 5′-CTGCTGCCTCCCGTAGGAGT-3′).

### 2.14. Statistical Analyses

Data are presented as mean ± SD. All statistical analyses were performed using GraphPad Prism 8 (GraphPad Software, San Diego, CA, USA). Differences between the two groups were evaluated using an unpaired two-tailed *t*-test. For comparisons among three or more groups, one-way ANOVA followed by Tukey’s post hoc test was applied. Statistically significant differences are indicated by asterisks as follows: * *p* < 0.05, ** *p* < 0.01, *** *p* < 0.001, and **** *p* < 0.0001; “ns” denotes non-significant comparisons.

## 3. Results

### 3.1. HTG Exacerbates Pancreatic Injury and Innate Immune Imbalance in AP

**Figure 1:**
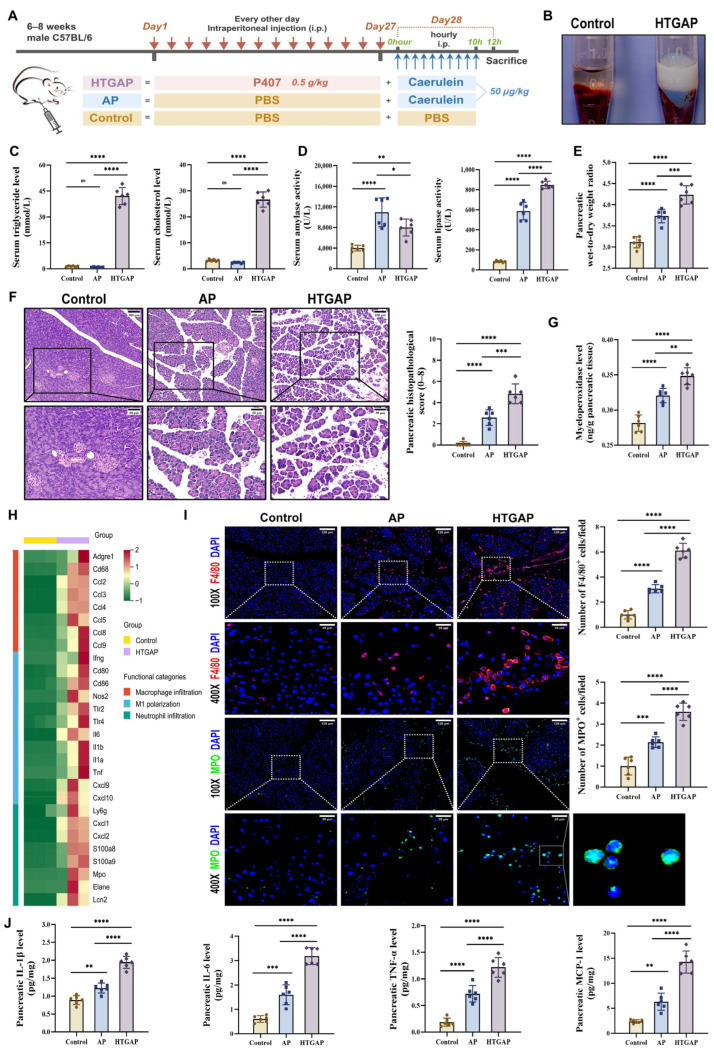
Hypertriglyceridemia (HTG) exacerbates pancreatic injury and innate immune imbalance in acute pancreatitis (AP). (**A**) Animal protocol. (**B**) Clear serum from control mice (left) and milky serum from HTGAP mice (right). (**C**) Serum triglyceride (TG) and total cholesterol (TC) levels (*n* = 6). (**D**) Serum amylase and lipase activities (*n* = 6). (**E**) Pancreatic wet-to-dry weight radio (*n* = 6). (**F**) Representative images of the pancreas by hematoxylin and eosin (H&E) staining and quantification of histology scores. Scale bars: 100 µm (200×) and 50 µm (400×) (*n* = 6). (**G**) Pancreatic myeloperoxidase (MPO) activity (*n* = 6). (**H**) Heatmap of differentially expressed genes identified by RNA-seq related to macrophage infiltration, M1 polarization, and neutrophil infiltration in pancreatic tissues between control and HTGAP mice (*n* = 3). (**I**) Representative immunofluorescence photomicrographs and quantification of pancreatic F4/80^+^ (red) and MPO^+^ (green) cells, expressed relative to control group means. Nuclei were stained with DAPI (blue). Scale bars: 120 µm (100×) and 30 µm (400×) (*n* = 6). (**J**) Pancreatic IL-1β, IL-6, TNF-α, and MCP-1 expressions tested by ELISA (*n* = 6). Data are presented as means ± SD from three independent experiments. * *p* < 0.05, ** *p* < 0.01, *** *p* < 0.001, **** *p* < 0.0001, and “ns” denotes not significant, as determined by one-way ANOVA followed by Tukey’s post hoc test for multiple comparisons.

In this study, we established the HTGAP mouse model following previously reported methods (Figure 1A) [3,4,6,21].

As shown in Figure 1B,C, a significant increase in serum chylomicrons, TG, and TC levels was observed in HTGAP mice. Notably, HTGAP mice exhibited remarkably elevated serum lipase but decreased serum amylase levels in comparison to AP mice (Figure 1D). The observed reduction in serum amylase likely reflects assay interference caused by severe HTG (serum TG > 5.65 mmol/L), which increases sample turbidity and leads to falsely low amylase measurements [27]. This phenomenon confirms that serum lipase serves as a more reliable diagnostic marker than amylase for HTGAP. Histopathological analysis demonstrated significantly greater pancreatic tissue damage in HTGAP mice relative to AP controls, characterized by more severe edema, inflammatory cell infiltration, and necrosis (Figure 1F). Moreover, the pancreatic wet-to-dry weight ratio (Figure 1E) was also significantly elevated in HTGAP mice compared with AP mice. Transcriptomic profiling of pancreatic tissues revealed enhanced macrophage infiltration, M1 polarization, and neutrophil infiltration in HTGAP mice compared to the control group (Figure 1H). Consistent with these findings, immunofluorescence staining confirmed elevated infiltration of both macrophages (F4/80^+^ cells) and neutrophils (MPO staining and activity) in HTGAP pancreatic tissue (Figure 1G,I). Furthermore, HTGAP mice exhibited significantly upregulated expression of pro-inflammatory cytokines (IL-1β, TNF-α, IL-6) and chemokines (MCP-1) in pancreatic tissue (Figure 1J). Collectively, these combined findings implied that HTG aggravates the pancreatic injury of AP.

### 3.2. HTGAP Exacerbates Colonic Barrier Damage and Pancreatic Bacterial Translocation

**Figure 2:**
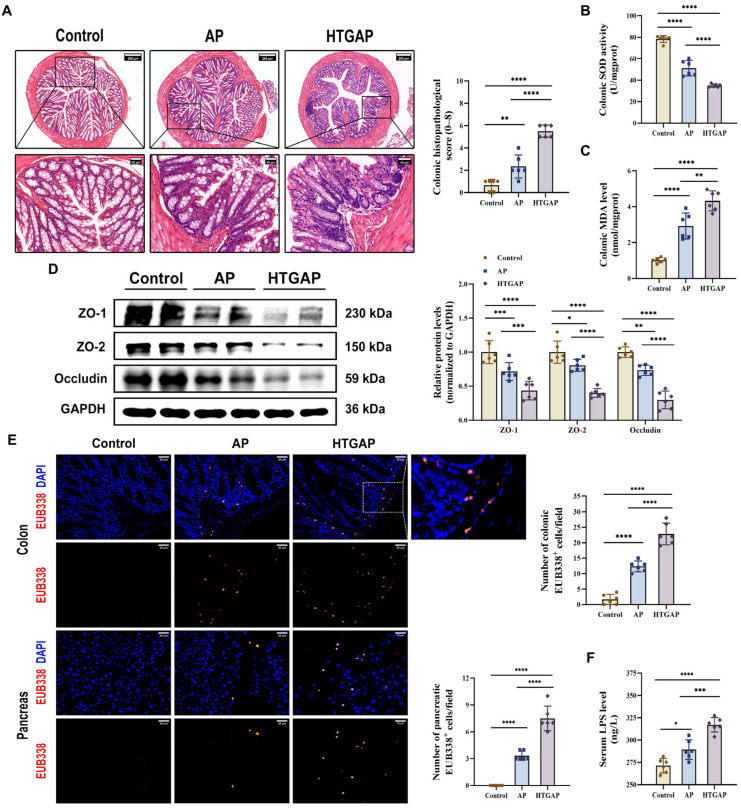
HTGAP exacerbates colonic barrier damage and bacterial translocation to the pancreas. (**A**) Representative images of the colon by H&E staining and quantification of histology scores. Scale bars: 200 µm (100×) and 40 µm (500×). (**B**) Colonic superoxide dismutase (SOD) activity. (**C**) Colonic malondialdehyde (MDA) levels. (**D**) Western blot and densitometry analyses of colonic tight junction proteins (TJPs) ZO-1, ZO-2, and Occludin, normalized to GAPDH. (**E**) Positive hybridization signals of total bacteria in the colon and pancreas detected by the FISH probe EUB338 (red). Nuclei were stained with DAPI (blue). Scale bar: 30 µm (400×). (**F**) Intestinal permeability was assessed by measuring serum lipopolysaccharide (LPS) levels using ELISA. Data are presented as means ± SD from three independent experiments. *n* = 6 per group. * *p* < 0.05, ** *p* < 0.01, *** *p* < 0.001, and **** *p* < 0.0001, as determined by one-way ANOVA followed by Tukey’s post hoc test for multiple comparisons.

Translocation of enteric bacteria and endotoxins to the pancreas due to compromised intestinal barrier integrity is a well-established mechanism that exacerbates AP [10]. Our study revealed that HTGAP induces severe colonic oxidative stress and barrier damage, characterized by increased colonic histopathological scores (Figure 2A), suppressed SOD antioxidant activity (Figure 2B), elevated MDA levels (Figure 2C), downregulated the expression of tight junction proteins (TJPs) (ZO-1, ZO-2, and Occludin) (Figure 2D), increased serum LPS levels (Figure 2F), and enhanced bacterial translocation to both intestinal mucosa and pancreatic tissue, as demonstrated by FISH staining (Figure 2E). Overall, our findings suggest that HTGAP exacerbates intestinal barrier disruption and bacterial translocation.

### 3.3. HTGAP Exacerbates Colonic Mucus Layer Disruption and M1 Macrophage Polarization

**Figure 3:**
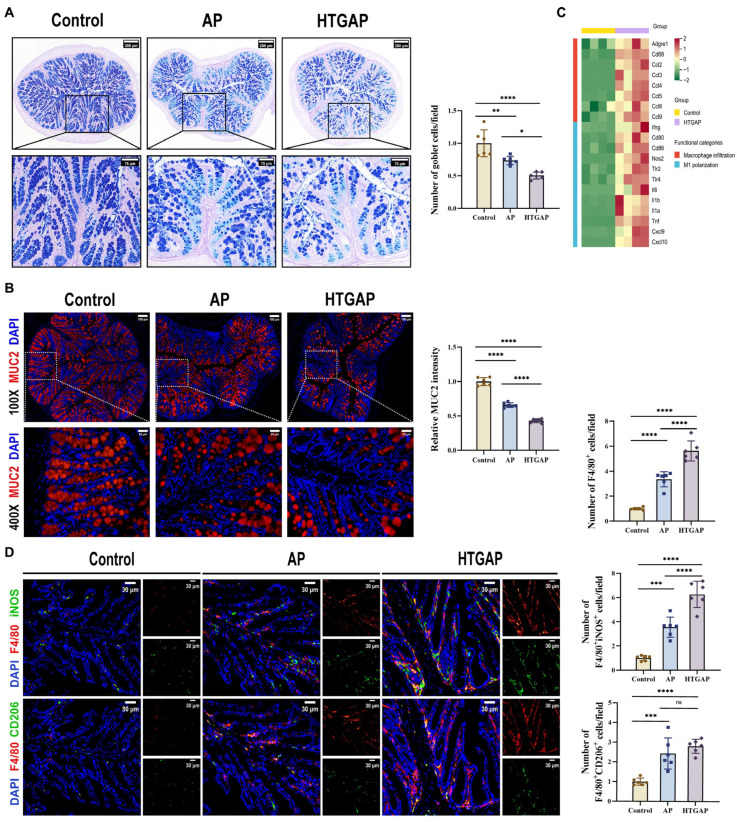
HTGAP exacerbates colonic mucus layer disruption and M1 macrophage polarization. (**A**) Representative images of Alcian Blue-Periodic Acid-Schiff (AB-PAS) staining of colonic tissues and corresponding quantitative analysis normalized to the control group means. Scale bar: 200 µm (115×) and 75 µm (350×) (*n* = 6). (**B**) Representative immunofluorescence photomicrographs and quantitative analysis of colonic MUC2 intensity (red), expressed relative to control group means. Nuclei were counterstained with DAPI (blue). Scale bar: 100 µm (100×) and 25 µm (400×) (*n* = 6). (**C**) Heatmap of differentially expressed genes identified by RNA-seq related to macrophage infiltration and M1 polarization in colonic tissues between control and HTGAP mice (*n* = 4). (**D**) Representative immunofluorescence photomicrographs and quantitative analysis of F4/80^+^iNOS^+^ cells (upper panels) and F4/80^+^CD206^+^ cells (lower panels) in colonic tissues, normalized to control group means. Red, F4/80; green, iNOS (upper) or CD206 (lower). Nuclei were stained with DAPI (blue). Scale bars: 30 µm (400×) (*n* = 6). Data are presented as means ± SD from three independent experiments. * *p* < 0.05, ** *p* < 0.01, *** *p* < 0.001, **** *p* < 0.0001, and “ns” denotes not significant, as determined by one-way ANOVA followed by Tukey’s post hoc test for multiple comparisons.

AB-PAS staining revealed a marked reduction in colonic goblet cells in HTGAP mice compared to AP controls (Figure 3A). This finding was corroborated by immunofluorescence staining, which showed that MUC2 was only modestly decreased in AP mice but was dramatically reduced in HTGAP mice (Figure 3B). Transcriptomic analysis demonstrated significant upregulation of genes associated with macrophage infiltration and M1 polarization in HTGAP colonic tissue (Figure 3C). Immunofluorescence staining further confirmed a substantially expanded population of infiltrating macrophages (F4/80^+^ cells) and M1 macrophages (F4/80^+^iNOS^+^) in the colonic tissue of HTGAP mice. By comparison, the number of M2 macrophages (F4/80^+^CD206^+^) showed no significant difference between the AP and HTGAP groups (Figure 3D). Taken together, these results suggest that HTGAP exacerbates gut barrier dysfunction by disrupting the mucus layer and M1 macrophage polarization.

### 3.4. Gut Dysbiosis Is Involved in the Severity of HTGAP

**Figure 4:**
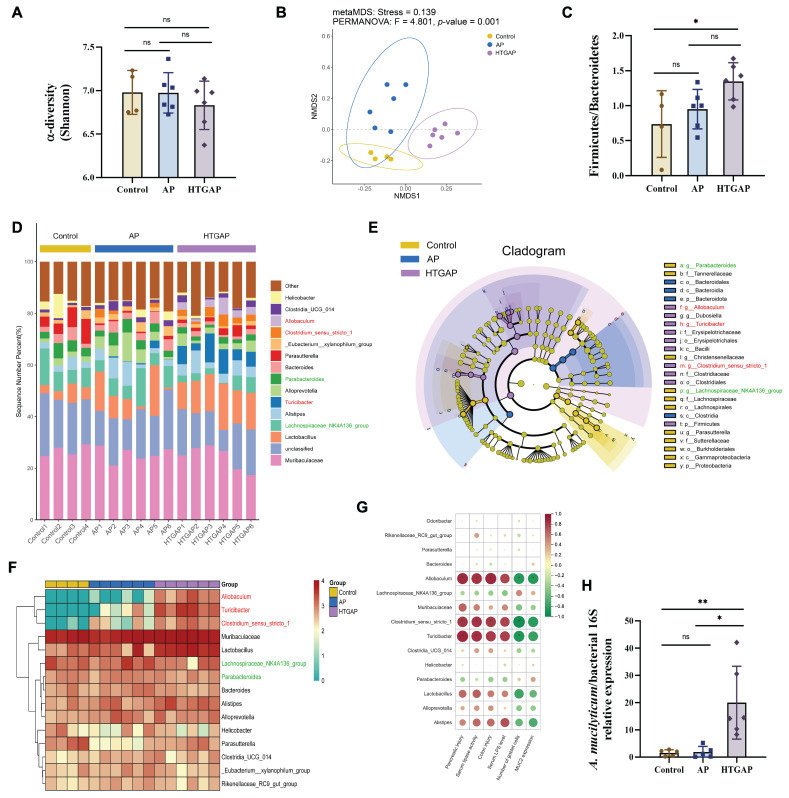
Gut dysbiosis is implicated in the progression of HTGAP. (**A**) Alpha diversity (α-diversity) analysis based on the Shannon index. (**B**) Non-metric multidimensional scaling (NMDS) plot from beta diversity (β-diversity) analysis based on the Bray–Curtis distance, illustrating distinct clustering patterns of microbial communities among groups. (**C**) Ratio of Firmicutes to Bacteroidetes in the gut microbiota. (**D**) Taxonomic composition at the genus level, with relative abundances of the top 15 genera shown. (**E**) Cladogram based on linear discriminant analysis effect size (LEfSe) showing characteristic differential bacterial taxa across groups. Taxonomic levels are indicated as follows: p, phylum; c, class; o, order; f, family; g, genus. (**F**) Hierarchical clustering heatmap at the genus level, showing the absolute abundances of the top 15 genera across groups. (**G**) Correlation heatmap generated by Spearman correlation analysis between disease phenotypic indicators and differential bacterial genera (* *p* < 0.0001). (**H**) The abundance of *Allobaculum mucilyticum* (*A. mucilyticum*) was determined by qPCR. In panels (**A**,**C**,**H**), data are presented as means ± SD from three independent experiments. * *p* < 0.05, ** *p* < 0.01, and “ns” denotes not significant, as determined by one-way ANOVA followed by Tukey’s post hoc test for multiple comparisons.

To elucidate the role of gut microbiota in pancreatic and colonic barrier injury in the HTGAP model, 16S rRNA sequencing was performed on colonic contents. The Shannon index indicated no significant differences in microbial α-diversity among groups (Figure 4A). In contrast, β-diversity analysis based on NMDS revealed distinct compositional differences (Figure 4B), with Bray–Curtis ANOSIM confirming clear separation between all groups: Control vs. AP (*p* = 0.035), Control vs. HTGAP (*p* = 0.005), and AP vs. HTGAP (*p* = 0.001). The Firmicutes/Bacteroidetes ratio, a phylum-level marker of dysbiosis [18], was significantly elevated in HTGAP mice (Figure 4C). The percentage-stacked bar chart in Figure 4D displays the relative abundances of the top 15 genera across groups. LEfSe analysis identified predominant and differentially abundant taxa in each group (Figure 4E). *Lachnospiraceae_NK4A136_group* and *Parabacteroides* were predominant in controls but modestly decreased in AP and HTGAP groups (Figure 4F). Conversely, *Allobaculum*, *Turicibacter*, and *Clostridium_sensu_stricto_1* were absent in controls, moderately increased in AP mice, and strikingly enriched in HTGAP mice, as shown by the clustering heatmap (Figure 4F). To identify specific bacterial taxa potentially contributing to HTGAP progression, correlations between gut microbiota composition and phenotypic indicators were assessed. *Allobaculum* abundance was most positively correlated with serum lipase, intestinal permeability, and pancreatic and colonic pathology, and most negatively associated with colonic goblet cell numbers and MUC2 expression levels (Figure 4G). Additionally, qPCR analysis validated the significant enrichment of the newly identified pathobiont *A. mucilyticum* in HTGAP mice (Figure 4H). In summary, HTGAP reshapes gut microbiota composition, characterized by enrichment of potential pathobionts rather than depletion of beneficial taxa, which may be involved in HTGAP progression.

### 3.5. Administration of A. mucilyticum Aggravates Gut Barrier Dysfunction and HTGAP Severity in Mice

**Figure 5:**
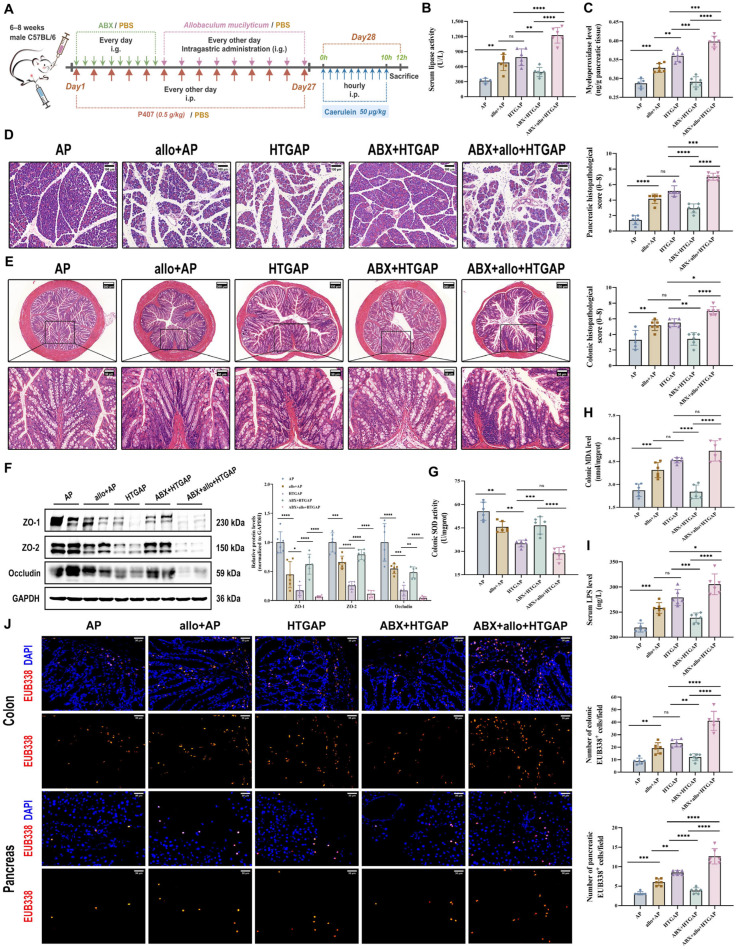
Administration of *A. mucilyticum* aggravates gut barrier dysfunction and HTGAP severity in mice. (**A**) Schematic diagram of *A. mucilyticum* supplementation protocol. (**B**) Serum lipase activity. (**C**) Pancreatic MPO activity. (**D**) Representative H&E-stained images of the pancreas and quantitative analysis of histological scores. Scale bar: 100 µm (200×). (**E**) Representative H&E-stained images of the colon and quantitative analysis of histological scores. Scale bars: 150 µm or 200 µm (135× or 100×) and 50 µm (400×). (**F**) Representative Western blot and densitometric analyses of colonic TJPs ZO-1, ZO-2, and Occludin normalized to GAPDH. (**G**) Colonic SOD activity. (**H**) Colonic MDA levels. (**I**) Serum LPS level. (**J**) Positive hybridization signals for total bacteria in the colon and pancreas were detected using the FISH probe EUB338 (red). Nuclei were counterstained with DAPI (blue). Scale bar: 30 µm (400×). Data are presented as means ± SD from three independent experiments. *n* = 6 per group. * *p* < 0.05, ** *p* < 0.01, *** *p* < 0.001, **** *p* < 0.0001, and “ns” denotes not significant, as determined by one-way ANOVA followed by Tukey’s post hoc test for multiple comparisons.

To determine whether *Allobaculum* influences the severity of HTGAP, mice were orally gavaged with the *A. mucilyticum* strain every other day for two weeks before AP induction (Figure 5A). In ABX-treated HTGAP mice, administration of *A. mucilyticum* exacerbated pancreatic inflammation, as demonstrated by elevated serum lipase levels (Figure 5B), increased pancreatic MPO activity (Figure 5C), and aggravated histological damage (Figure 5D). Similarly, *A. mucilyticum* treatment worsened colonic pathology (Figure 5E), downregulated TJPs (ZO-1, ZO-2, and Occludin; Figure 5F), excessive colonic oxidative stress (Figure 5G, H), elevated serum LPS (Figure 5I), and promoted bacterial translocation to the intestinal mucosa and pancreas (Figure 5J). Consistent with prior findings, ABX pretreatment significantly attenuated HTGAP severity (Figure 5A–J). In addition, *A. mucilyticum* supplementation also intensified pancreatic damage and intestinal barrier dysfunction in AP mice (Figure 5A–J). Collectively, these results suggest that gut dysbiosis-driven *A. mucilyticum* exacerbates pancreatic injury, likely through disruption of intestinal barrier integrity.

### 3.6. Administration of A. mucilyticum Aggravates Colonic Mucus Layer Disruption and Immune Dysregulation

**Figure 6:**
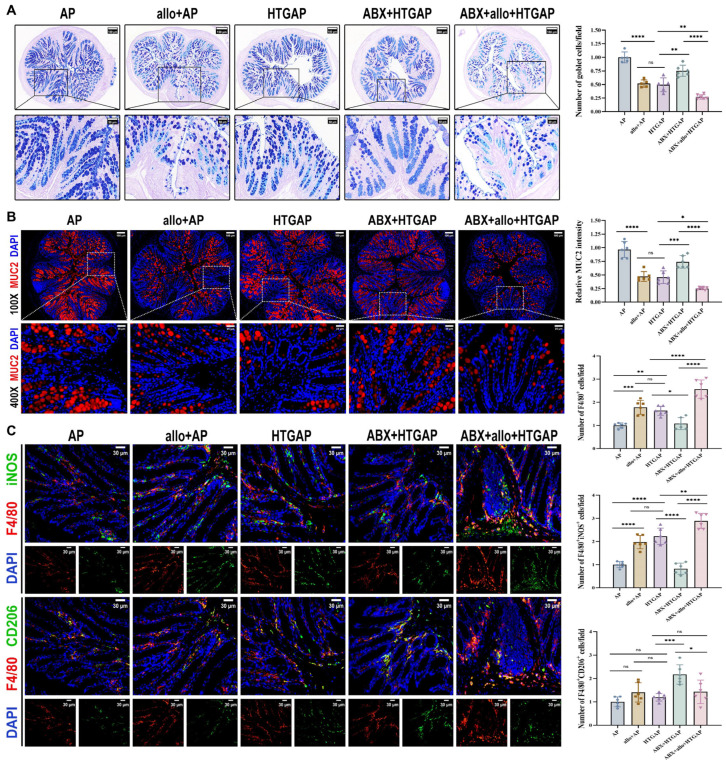
Administration of *A. mucilyticum* aggravates colonic mucus layer disruption and immune dysregulation. (**A**) Representative images of AB-PAS staining of colonic tissues and corresponding quantitative analysis normalized to the AP group means. Scale bars: 150 µm or 200 µm (low magnification) and 50 µm (400×). (**B**) Representative immunofluorescence photomicrographs and quantitative analysis of colonic MUC2 intensity (red), expressed relative to AP group means. Nuclei were stained with DAPI (blue). Scale bars: 100 µm (100×) and 25 µm (400×). (**C**) Representative immunofluorescence photomicrographs and quantitative analysis of F4/80^+^iNOS^+^ cells (upper panels) and F4/80^+^CD206^+^ cells (lower panels) in colonic tissues, normalized to AP group means. Red, F4/80; green, iNOS (upper) or CD206 (lower). Nuclei were counterstained with DAPI (blue). Scale bars: 30 µm (400×). Data are presented as means ± SD from three independent experiments. *n* = 6 per group. * *p* < 0.05, ** *p* < 0.01, *** *p* < 0.001, **** *p* < 0.0001, and “ns” denotes not significant, as determined by one-way ANOVA followed by Tukey’s post hoc test for multiple comparisons.

*A. mucilyticum*, which encodes numerous mucin-degrading enzymes (CAZymes), promotes mucus layer degradation and triggers inflammatory responses that contribute to intestinal barrier dysfunction [24,28,29,30,31]. Supplementation with *A. mucilyticum* significantly reduced colonic goblet cell numbers (Figure 6A) and MUC2 expression (Figure 6B), while increasing total macrophage infiltration (F4/80^+^ cells) and M1 macrophage polarization (F4/80^+^iNOS^+^ cells) in ABX-treated HTGAP mice (Figure 6C). In contrast, ABX treatment effectively restored colonic goblet cell numbers (Figure 6A) and MUC2 expression (Figure 6B), while suppressing macrophage infiltration and M1 polarization in HTGAP mice (Figure 6C). Similarly, in AP mice, *A. mucilyticum* supplementation decreased goblet cell numbers (Figure 6A) and MUC2 expression (Figure 6B), while enhancing macrophage infiltration and M1 polarization (Figure 6C). Together, these findings demonstrate that *A. mucilyticum* exacerbates intestinal barrier dysfunction, at least in part, by compromising the mucus barrier and promoting local inflammation.

### 3.7. A. muciniphila Repairs the Colonic Barrier and Rebalances Immunity

**Figure 7:**
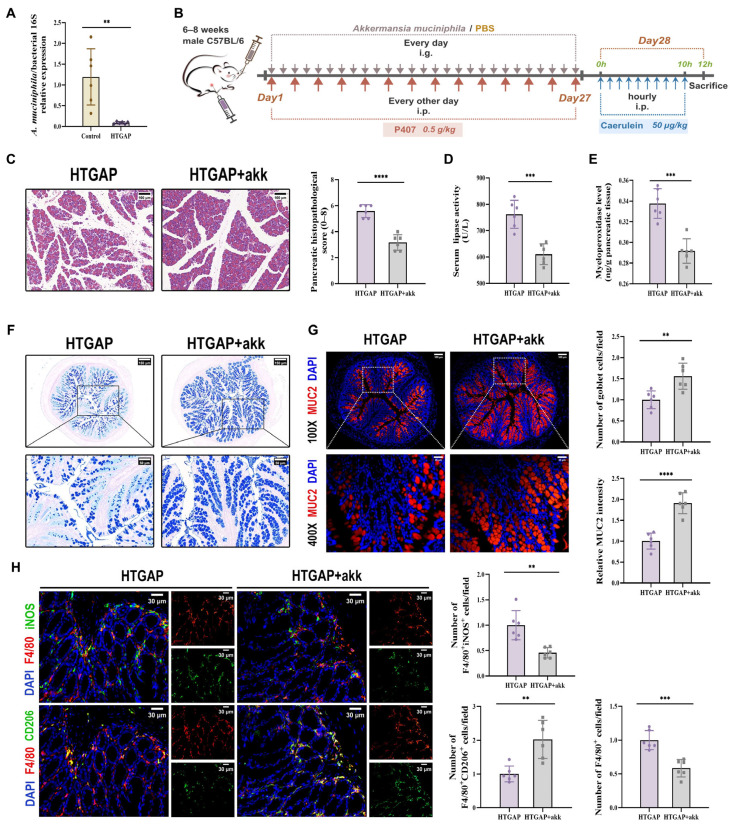
*Akkermansia muciniphila (A. muciniphila)* alleviates pancreatic injury in HTGAP via mucus layer restoration and immune modulation. (**A**) The abundance of *A. muciniphila* was tested by qPCR. (**B**) Schematic representation of the experimental design for *A. muciniphila* supplementation. (**C**) Representative H&E-stained images of the pancreas and quantitative analysis of histological scores. Scale bar: 100 µm (200×). (**D**) Serum lipase activity. (**E**) Pancreatic MPO activity. (**F**) Representative images of AB-PAS staining of colonic tissues and corresponding quantitative analysis normalized to the HTGAP group means. Scale bars: 150 µm (130×) and 50 µm (400×). (**G**) Representative immunofluorescence photomicrographs and quantitative analysis of colonic MUC2 intensity (red), expressed relative to HTGAP group means. Nuclei were stained with DAPI (blue). Scale bars: 100 µm (100×) and 25 µm (400×). (**H**) Representative immunofluorescence photomicrographs and quantitative analysis of F4/80^+^iNOS^+^ cells (upper panels) and F4/80^+^CD206^+^ cells (lower panels) in colonic tissues, normalized to HTGAP group means. Red, F4/80; green, iNOS (upper) or CD206 (lower). Nuclei were stained with DAPI (blue). Scale bars: 30 µm (400×). Data are presented as means ± SD from three independent experiments. *n* = 6 per group. ** *p* < 0.01, *** *p* < 0.001, and **** *p* < 0.0001, as determined by unpaired two-tailed *t*-test.

*A. muciniphila* has been previously reported to restore mucus layer thickness and modulate immune responses [24,26,32]. Although *A. muciniphila* has shown beneficial effects in AP, its abundance is significantly reduced in HTGAP [20,21]. Consistent with previous findings, we observed decreased *A. muciniphila* abundance in our HTGAP model (Figure 7A). To investigate the regulatory role of *A. muciniphila* in gut barrier integrity and HTGAP severity, we administered *A. muciniphila* to HTGAP mice (Figure 7B).

**Figure 8:**
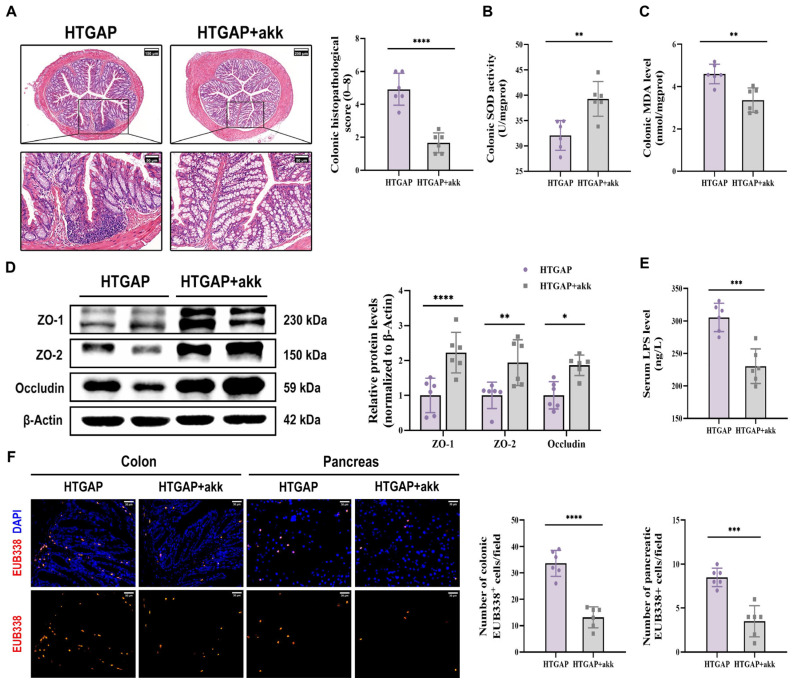
*A. muciniphila* reinforces the colonic barrier and limits bacterial translocation. (**A**) Representative H&E-stained images of the colon and quantitative analysis of histological scores. Scale bars: 150 µm or 200 µm (140× or 105×) and 50 µm (400×). (**B**) Colonic SOD activity. (**C**) Colonic MDA levels. (**D**) Western blot and densitometric analyses of colonic TJPs ZO-1, ZO-2, and Occludin normalized to β-Actin. (**E**) Serum LPS level. (**F**) Positive hybridization signals of total bacteria in the colon and pancreas were detected using the FISH probe EUB338 (red). Nuclei were counterstained with DAPI (blue). Scale bar: 30 µm (400×). Data are presented as means ± SD from three independent experiments. *n* = 6 per group. * *p* < 0.05, ** *p* < 0.01, *** *p* < 0.001, and **** *p* < 0.0001, as determined by unpaired two-tailed *t*-test.

Treatment with *A. muciniphila* significantly ameliorated pancreatic pathological lesions, reduced serum lipase levels, and decreased pancreatic MPO activity (Figure 7C–E), confirming its protective effects. Furthermore, *A. muciniphila* administration restored colonic goblet cell numbers (Figure 7F) and MUC2 expression (Figure 7G), while attenuating macrophage infiltration and M1 polarization (Figure 7H). Notably, *A. muciniphila* substantially improved intestinal barrier function in HTGAP mice, as evidenced by reduced colonic histological scores (Figure 8A), ameliorated colonic oxidative stress (Figure 8B, C), upregulated TJPs (ZO-1, ZO-2, and Occludin) expression (Figure 8D), decreased serum LPS levels (Figure 8E) and inhibited bacterial translocation to both intestinal mucosa and pancreas (Figure 8F). Collectively, these findings demonstrate that *A. muciniphila* supplementation effectively attenuates HTGAP severity.

## 4. Discussion

Our findings indicate that gut microbiota dysbiosis, particularly the abnormal enrichment of the pathogenic immunostimulatory bacterium *A. mucilyticum*, aggravates the progression of HTGAP. *A. mucilyticum*-mediated degradation of the colonic mucus layer facilitates the adhesion of harmful bacteria to the intestinal epithelium, thereby triggering M1 macrophage polarization in the lamina propria and the release of pro-inflammatory cytokines, which together compromise intestinal barrier integrity. The resultant increase in intestinal permeability promotes the translocation of bacteria and endotoxins to the pancreas, ultimately resulting in pancreatic infection and enhanced infiltration of pancreatic M1 macrophages. In contrast, supplementation with *A. muciniphila* restores colonic barrier function, protecting against the development of HTGAP.

HTGAP is more prone to progress into critical illness with mortality rates as high as 20–40% [6]. Consistent with previous reports [3,4,5,21], our data support that HTG exacerbates pancreatic injury in AP and promotes pancreatic innate immune cell infiltration. HTGAP results in more serious systemic complications, including IPN, than other types of AP [7,8,9]. Intestinal flora disorders, gut barrier damage, mucosal immune imbalance, and translocation of enteropathogenic bacteria are the main causes of IPN in severe AP [10,13,14,15,16,17,18,33,34,35,36]. HTGAP patients harbor remarkably altered intestinal community structures, characterized by enrichment of pathogenic bacteria and depletion of protective taxa. These variations are strongly correlated with clinical indicators of disease severity and unfavorable prognosis [20,21]. Fecal microbiota transplantation (FMT) from HTGAP patients exhibited worsened pancreatic injury and systemic inflammation in mice compared to FMT from healthy volunteers or other AP etiologies, demonstrating that HTG-remodeled microbial composition alterations contribute to disease aggravation [21]. Additionally, pancreatic and ileal histopathological injuries in HTGAP may be attributed to the reduced secretion of AMPs by Paneth cells due to gut dysbiosis [19].

The colonic mucus layer, maintained by continuous replenishment of mucins synthesized by goblet cells, fills the crypts and covers the apical surface of the intestinal epithelial monolayer, restricting direct contact between luminal bacteria and epithelial or immune cells in the lamina propria [37,38]. Mice lacking the predominant secretory mucin MUC2 develop spontaneous colitis [39]. Consistent with earlier findings that western-style diet-driven gut dysbiosis, especially enrichment of mucus-degrading microbiota, causes functional defects in the inner colonic mucus layer by reducing mucus growth rate and increasing permeability [40]. The mucus layer is thinning in HTGAP mice, allowing numerous bacteria to penetrate deeper into the crypts and colonize the colonic mucosa and submucosa, thereby stimulating pro-inflammatory macrophage overactivation, which results in crypt loss and barrier dysfunction.

Our microbial 16S rRNA sequencing revealed that *Allobaculum* was one of the most affected genera by HTGAP. Its expansion coincided with the destruction of the mucus layer and was positively correlated with colonic and pancreatic lesions. Marked enrichment of *Allobaculum* has also been observed in gut microbiota data from multiple studies on HTGAP, high-fat diet-induced obesity, type 2 diabetes, and fatty acid or lipid metabolism [19,40,41,42,43,44,45]. *A. mucilyticum*, newly isolated from ulcerative colitis patients and rarely present in healthy individuals, degrades mucins such as MUC2 by secreting various types of mucin O-glycans-targeting CAZymes, and utilizes the released monosaccharides as substrates for colonization and proliferation [31]. Colonization with *A. mucilyticum* in germ-free mice reduced mucus thickness and facilitated bacterial encroachment into epithelial ecological niches [24]. Specific bacterial species and related metabolites have been shown to alter the progression of parenteral diseases, including HTGAP, by influencing intestinal immunity [10,11,12,17,21,46]. Besides its mucus-degrading properties, *A. mucilyticum* is considered a unique colitogenic taxon due to its pathogenic immunostimulatory effects, which significantly upregulate genes involved in inflammatory cytokine production, leukocyte proliferation, and immune activation [24,29]. In a study on low-dose antibiotic exposure-induced metabolic alterations, *Allobaculum* abundance correlated positively with ileal Th17 transcription factor RORγT and IL-17 expression, suggesting a role in intestinal Th17 differentiation [28]. *Allobaculum* strains induce the production of epithelial-derived serum amyloid A and dendritic cell-derived IL-23 by adhering to epithelial cells, thereby activating antigen-specific pathogenic Th17 cells in the small intestine and triggering robust EAE symptoms [30].

Interestingly, we observed an inverse correlation between the abundances of *Allobaculum* and *A. muciniphila* in multiple investigations of high-fat-induced gut microbiota alterations [24,40,41,45]. *A. muciniphila* was more prevalent in healthy populations and less common in HTGAP patients. Its abundance was negatively correlated with HTGAP severity and poor prognosis [20,21]. Our experimental data yielded the same outcomes. *A. muciniphila* resides in the extracolonic mucus layer, occupying ecological habitats and preventing the invasion and adhesion of other pathogens to intestinal epithelial cells [37]. They utilize O-glycans without compromising mucin network integrity and can compensate by boosting mucus secretion, thus preserving the dynamic equilibrium of mucus layer renewal [26]. In addition, *A. muciniphila* exerts immunomodulatory effects by promoting macrophage polarization toward the anti-inflammatory, tissue-repairing M2 phenotype, while limiting the proliferation of pro-inflammatory CD16/32^+^ M1 macrophages and the generation of pro-inflammatory substances [32]. Our results demonstrated that *A. muciniphila* colonization ameliorates mucus damage and pathological intestinal innate immune responses incited by *A. mucilyticum*, decreasing intestinal inflammation and bacterial translocation, ultimately mitigating HTGAP-associated pancreatic injury. These findings align with previous studies on DSS-induced colitis, where *A. muciniphila* and *Allobaculum* elicited opposing immunological effects and competitively suppressed each other’s immune responses [24]. A possible explanation for this might be that individuals possess only a handful of potent immunostimulatory strains, which may compete for limited unique immunogenic niches.

Overall, we have identified HTG-induced expansion of the pro-inflammatory mucolytic pathobiont *A. mucilyticum* as a key contributor to pancreatic injury via gut barrier disruption. This discovery broadens our understanding of the gut-pancreas axis in HTGAP by linking gut microbiota dysbiosis and mucosal immune alterations to worsened pancreatic inflammation. Nevertheless, this study has certain limitations. We employed the widely accepted and best-validated HTGAP mouse model, which is effective for exploring pathogenesis. However, as with all animal models, it may not fully reflect the complex pathophysiology of human HTGAP. Therefore, future studies involving patient samples or translational models would be valuable for clinical validation. In addition, our results showed that probiotic *A. muciniphila* partially alleviates mucus degradation and pro-inflammatory immune activation triggered by pathobiont *A. mucilyticum*. These findings provide a rational basis for formulating microbiota-targeted strategies in HTGAP. Given that early enteral nutrition (EN) is widely recommended in current clinical guidelines for managing AP to preserve gut homeostasis and reduce infectious complications, further research could incorporate multi-omics analyses, including metabolomics and pathway-specific investigations, to identify suitable microbial metabolites for EN and to explore their molecular mechanisms in influencing host responses.

## 5. Conclusions

**Figure 9:**
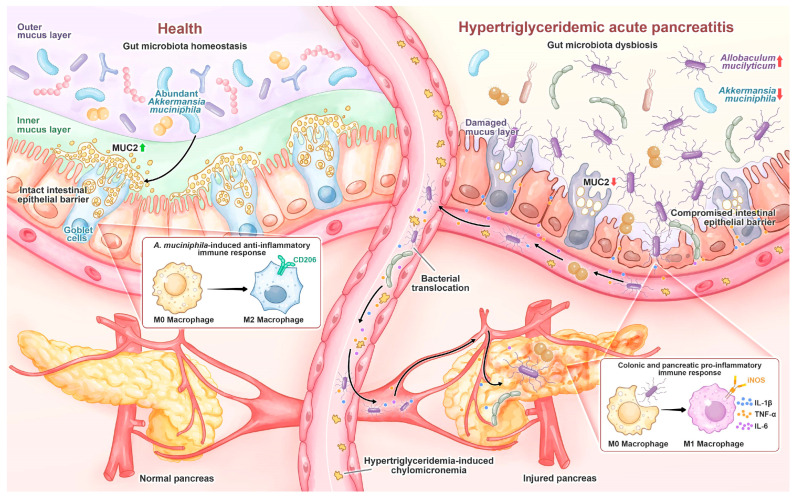
In healthy individuals, *A. muciniphila* is prevalent and maintains gut homeostasis by stimulating goblet cell MUC2 production and promoting M2 macrophage polarization. During HTGAP, gut microbiota dysbiosis, characterized by the reduction in *A. muciniphila* and enrichment of *A. mucilyticum*, disrupts the colonic mucus barrier and induces M1 macrophage polarization, leading to increased intestinal permeability and bacterial translocation to the pancreas, which aggravates pancreatic damage.

In the murine HTGAP model, HTG-driven gut dysbiosis with expansion of *A. mucilyticum* disrupted the colonic mucus layer, thereby increasing intestinal permeability and enabling microbial migration to the pancreas, which ultimately aggravated pancreatic injury (Figure 9). Supplementation with *A. muciniphila* reversed these changes, suggesting that modulation of gut microbiota may represent a potential therapeutic approach.

## Figures and Tables

**Table 1:** Primary antibodies.

Antibody Name	Vendor	Catalog Number/Clone ID	Research Resource Identifier (RRID)
Beta Actin Mouse mAb	Proteintech	Cat# 66009-1-Ig; Clone ID: 2D4H5	AB_2687938
GAPDH Mouse mAb	Proteintech	Cat# 60004-1-Ig; Clone ID: 1E6D9	AB_2107436
ZO-1 Rabbit pAb	Proteintech	Cat# 21773-1-AP	AB_10733242
ZO-2 Rabbit pAb	Proteintech	Cat# 18900-1-AP	AB_2203584
Occludin Mouse mAb	Proteintech	Cat# 66378-1-Ig; Clone ID: 1D3C4	AB_2881755
MUC2 Rabbit mAb	Abcam	Cat# ab272692; Clone ID: EPR23479-47	AB_2888616
F4/80 Rabbit mAb	Cell Signaling Technology	Cat# 70076; Clone ID: D2S9R	AB_2799771
NOS2/iNOS Mouse mAb	Santa Cruz Biotechnology	Cat# sc-7271; Clone ID: C-11	AB_627810
MMR/CD206 Goat pAb	R&D Systems	Cat# AF2535	AB_2063012
Myeloperoxidase/MPO Goat pAb	R&D Systems	Cat# AF3667	AB_2250866

Antibodies were obtained from: Proteintech (Wuhan, China), Abcam (Cambridge, UK), Cell Signaling Technology (Danvers, MA, USA), Santa Cruz Biotechnology (Dallas, TX, USA), and R&D Systems (Minneapolis, MN, USA).

**Table 2:** Secondary antibodies.

Antibody Name	Vendor	Catalog Number	RRID
Goat Anti-Mouse IgG(H+L), HRP-conjugate	Proteintech	Cat# SA00001-1	AB_2722565
Goat Anti-Rabbit IgG(H+L), HRP-conjugate	Proteintech	Cat# SA00001-2	AB_2722564
Donkey Anti-Goat IgG(H+L), FITC-conjugate	Proteintech	Cat# SA00003-3	AB_2857365
Donkey Anti-Rabbit IgG (H+L), Cy3-conjugate	Servicebio	Cat# GB21403	AB_2818951
Goat Anti-rabbit IgG (H+L), F(ab’)2 Fragment (Alexa Fluor^®^ 555 Conjugate)	Cell Signaling Technology	Cat# 4413	AB_10694110
Goat Anti-Mouse IgG (H+L) Highly Cross-Adsorbed, Alexa Fluor™ Plus 488	Thermo Fisher Scientific	Cat# A32723	AB_2633275

Antibodies were obtained from: Proteintech (Wuhan, China), Servicebio (Wuhan, China), Cell Signaling Technology (Danvers, MA, USA), and Thermo Fisher Scientific (Waltham, MA, USA).

## Data Availability

The data supporting the findings of this study are available within the article. Sequencing data have been deposited in the NCBI Sequence Read Archive (PRJNA1303436, https://www.ncbi.nlm.nih.gov/bioproject/PRJNA1303436). Further information or requests for resources or reagents should be directed to the corresponding author.

## Abbreviations

The following abbreviations are used in this manuscript:

HTGAP	hypertriglyceridemic acute pancreatitis
AP	acute pancreatitis
HTG	hypertriglyceridemia
IPN	infected pancreatic necrosis
SIR	systemic inflammatory response
MOF	multiple organ failure
LPS	lipopolysaccharide
AMPs	antimicrobial peptides
ABX	broad-spectrum antibiotic
P407	poloxamer 407
CER	caerulein
PBS	phosphate-buffered saline
TG	triglyceride
TC	total cholesterol
MPO	myeloperoxidase
H&E	hematoxylin and eosin
RT	room temperature
ELISA	enzyme-linked immunosorbent assay
FISH	fluorescence in situ hybridization
SOD	superoxide dismutase
MDA	malondialdehyde
AB-PAS	Alcian Blue-Periodic Acid-Schiff
ASVs	amplicon sequence variants
NMDS	non-metric multidimensional scaling
LEfSe	linear discriminant analysis effect size
CAZymes	mucin-degrading enzymes
TJPs	tight junction proteins
FMT	fecal microbiota transplantation
RRID	Research Resource Identifier
EN	enteral nutrition
i.p.	intraperitoneal injection
i.g.	intragastric administration
